# Society Meetings

**Published:** 1906-08

**Authors:** 


### SOCIETY MEETINGS.

The American Association of Obstetricians and Gynecologists will hold its nineteenth annual meeting at the Hotel Havlin, Vine Street, between Fifth and Sixth Streets, Cincinnati, Thursday, Friday and Saturday, September 20, 21 and 22, 1906.

The Fellows of the association residing in Cincinnati constitute the committee of arrangements, of which Dr. Charles Lybrand Bonifield, 432 West Fourth Street, is the chairman, and Dr. Magnus A. Tate, Vindondissa Building, is the secretary, either of whom will furnish information to members and guests upon application.

The following is a list of papers offered up to the present date:

1. The president's address, John Young Brown, Saint Louis.
2. Porro-Cesarean section for pregnancy complicating fibroids; mother and child saved, James F. Baldwin, Columbus.
3. Fixation of the kidney by shortening of the nephrocolic ligament; with report of cases, Charles A. L. Reed, Cincinnati.
4. Obstruction of the bowel from gallstone, Miles F. Porter, Fort Wayne.
5. Preservation of the vault of the vagina in pelvic operations, A. Vander Veer, Albany.
6. Title to be announced, James N. West, New York.
7. Diffuse peritonitis, John B. Murphy, Chicago.
8. Title to be announced, Richard Douglas, Nashville.
9. Abuse of purgatives in surgical patients, Edwin Walker, Evansville.
10. Atresia vaginæ; observations and experience on symptoms and treatment, Augustus P. Clarke, Cambridge.
11. Title to be announced, J. H. Carstens, Detroit.
12. " " " " M. Rosenwasser, Cleveland.
13. Intestinal obstruction; report of cases, Rufus B. Hall, Cincinnati.
14. Vaginal celiotomy; its indications and limitations, Samuel Wyllis Bandler, New York.

15. The rectum in pelvic disease, H. O. Pantzer, Indianapolis.
16. The Gilliam operation for displacement of the uterus, D. Tod Gilliam, Columbus.
17. Title to be announced, X. O. Werder, Pittsburg.
18. " " " " Louis Frank, Louisville.
19. " " " " Walter B. Dorsett, Saint Louis.
20. " " " " W. A. B. Sellman, Baltimore.
21. " " " " Charles G. Cumston, Boston.
22. " " " " Wm. H. Wenning, Cinncinnati.
23. Report of a case, William H. Humiston, Cleveland.
24. Reflex dyspepsias from abdominal and pelvic disease, H. E. Hayd, Buffalo.
25. Peritoneal adhesions, Robert T. Morris, New York.
26. Kidney and colon suspension by use of the gastric capsule and nephrocolic ligament, Howard W. Longyear, Detroit.
27. Title to be announced, John D. S. Davis, Birmingham.
28. " " " " Walter B. Chase, Brooklyn.
29. " " " " Thomas B. Eastman, Indianapolis.
30. " " " " O. H. Elbrecht, Saint Louis.
31. Trend of the times in appendectomy, N. Stone Scott, Cleveland.
32. Puncture of the nonpuerperal uterus with prolapse of the bowel; violent seizure and tearing away of sixteen inches of gut; prompt abdominal section, intestinal anastamosis, and repair of uterine lacerations; recovery, Charles E. Congdon, Buffalo.
33. Epithelioma of the female urethra; a clinical note, L. S. McMurtry, Louisville.
34. Title to be announced, Joseph Price, Philadelphia.

John Young Brown,

President.

William Warren Potter,

Secretary.

---

The Buffalo Academy of Medicine is inviting all members to contribute to the program for the academic year beginning October 1, 1906. The title of the paper and the name of the section before it which it is proposed to read it should be sent to the general secretary, Dr. Edwin A. Bowerman, 234 E. Utica Street, Buffalo.

---

The British Medical Association will hold its seventy-fourth annual meeting at Toronto, August 21-25, 1906, under the administration of George Cooper Franklin, of Leicester, president, and Richard Andrews Reeve, of Toronto, president-elect.

THE MEDICAL SOCIETY OF THE COUNTY OF NIAGARA held its annual banquet at Olcott Beach Hotel, July 3, 1906. Dr. Roswell Park, of Buffalo, was the principal speaker and his subject was, "The Discovery of the Circulation."

---

THE LAKE KEUKA MEDICAL ASSOCIATION at its annual meeting held at Grove Springs, July 5-7, 1905, elected the following named officers for the ensuing year: president, J. E. Walker, Hornell; vice-president, F. A. Driesbach, Dansville; secretary and treasurer, H. B. Nichols, Pulteney.

---

THE AMERICAN ROENTGEN RAY SOCIETY will hold its seventh annual meeting at the Cataract-International Hotel, Niagara Falls, August 29-31, 1906, under the presidency of Dr. Henry Hulst, Grand Rapids, Mich. The secretary is Dr. George C. Johnston, 611 Fulton Building, Pittsburg.

